# Pharmacologic Interventions in Preventing Ovarian Hyperstimulation Syndrome: A Systematic Review and Network Meta-Analysis

**DOI:** 10.1038/srep19093

**Published:** 2016-01-11

**Authors:** Jun-Liang Guo, Duo-Duo Zhang, Yue Zhao, Dan Zhang, Xi-Meng Zhang, Can-Quan Zhou, Shu-Zhong Yao

**Affiliations:** 1 Department of Obstetrics & Gynaecology, The First Affiliated Hospital of Sun Yat-sen University, Guangzhou City, 510080, P.R. China; 2 Zhong-shan School of Medicine, Sun Yat-sen University, Guangzhou City, 510082, P.R. China

## Abstract

Ovarian hyperstimulation syndrome (OHSS) is a severe iatrogenic complication of controlled ovarian stimulation. Randomised controlled trials (RCTs) have proven several pharmacologic interventions to be effective in OHSS prevention, but these trials have seldom compared multiple drugs. We identified randomised controlled trials (RCTs) through June 2015 by searching databases and compared 11 intervention strategies in preventing OHSS (primary outcome) and their influence on pregnancy rate (secondary outcome). A network meta-analysis was used to evaluate the relative effectiveness among treatments and to create a rank probability table. Thirty-one RCTs were identified, including 7181 participants. Five pharmacologic interventions were superior to placebo in decreasing OHSS incidence: aspirin [relative risk (RR) 0.07, 95% credible interval (CrI) 0.01–0.30, *p* < 0.05], intravenous (IV) calcium [RR 0.11, 95% CrI 0.02–0.54, *p* < 0.05], cabergoline [RR 0.17, 95% CrI 0.06–0.43, *p* < 0.05], metformin [RR 0.20, 95% CrI 0.07–0.59, *p* < 0.05] and IV hydroxyethyl starch (HES) [RR 0.26, 95% CrI 0.05–0.99, *p* < 0.05]. The rank probability demonstrated as*p*irin (Rank 1: 36%) and IV calcium (Rank 1: 35%) to be the most efficacious. Additionally, albumin might decrease the pregnancy rate when compared with placebo [RR 0.85, 95% CI 0.74–0.97, *p* < 0.05]. This conclusion provides a relative standard and objective reference for choosing an OHSS prophylactic agent.

Ovarian hyperstimulation syndrome (OHSS) is an iatrogenic complication that arises during the processes of controlled ovarian stimulation (COS). As the most severe complication of COS, OHSS is associated with various signs and symptoms, including shortness of breath, abdominal distension, pleural effusion, ascites, and oedema of the extremities. The pathogenesis of OHSS has not been explained clearly until now. A common consensus demonstrates that OHSS is caused by the excessive secretion of the vasoactive agent vascular endothelial growth factor (VEGF) triggered by the use of an hCG trigger. VEGF causes a shift of fluid from the intravascular space to the third space and a subsequent haemoconcentration^1^. OHSS is a relatively dangerous disease due to the potential risk of thromboembolic diseases. The overall incidence of OHSS is 1–14% in all IVF/ICSI cycles^2^. According to a WHO report, the incidence of moderate and severe OHSS was 3–6% and 0.2–1%, respectively^3^. This disease imposes a heavy physical, psychological and economical burden on patients as a consequence of hospitalization, fear of infertility or miscarriage, and absenteeism.

Multiple interventions have been attempted to prevent OHSS from occurring in clinical trials, such as coasting^4^ and GnRH-antagonist protocol using a GnRh-agonist trigger^5^. However, these methods often require experienced clinicians and close monitoring during the COS process and may cause a decreased number of oocyte pick-ups^5^,^6^.

Pharmacologic interventions, which are relatively easily achievable and cost-effective, have been studied to decrease the incidence of OHSS. A series of randomized controlled trials and meta-analyses compared the effectiveness of different drugs. Leitao *et al.*^7^ showed that cabergoline reduces the incidence of moderate-severe OHSS compared with other drugs [relative risk (RR) 0.38, 95% confidence interval (CI) 0.29–0.51, 7 studies, 858 women] in a pair-wise meta-analysis. A Cochrane meta-analysis of RCTs claimed that metformin is effective in preventing OHSS^8^. Randomised controlled trials (RCTs) have provided evidence of other commonly used drugs with the aim of preventing OHSS. Intravenous (IV) calcium was demonstrated to be effective in a placebo-controlled trial reported by El-Khayat *et al.*^9^. Gokman *et al.*^10^ demonstrated that the administration of albumin and hydroxyethyl starch (HES) might prevent moderate to severe OHSS in high-risk patients. Nevertheless, few studies have compared multiple drugs. Clinicians typically depend on multiple treatment comparisons to make clinical decisions. Therefore, a lack of a direct comparison among drugs may lead to a clinical dilemma regarding medication options.

Network meta-analysis (NMA), which is a statistical method, enables a comparison among multiple interventions by synthesizing direct and indirect evidence from RCTs. Additionally, with a Bayesian approach, NMA is able to estimate a rank probability that can provide a rough rank of use of the interventions to guide clinical medication. Indirect treatment comparison (ITC) is used to compare two interventions indirectly from a common middle intervention if the direct comparison is unavailable. For example, if we have RCT(s) between interventions A and C and between interventions B and C, the ITC method can be applied to compare the effectiveness of A and B through the “bridge” comparator C.

In this article, we performed Bayesian NMA to determine the combined effectiveness of commonly used pharmacologic interventions (cabergoline, IV albumin, IV calcium infusion, IV HES infusion, aspirin, glucocorticoid, metformin) in preventing COS-related OHSS and to determine a treatment’s influence on pregnancy outcome. We also introduced a pair-wise meta-analysis for the comparison of direct evidence and ITC for indirect evidence.

## Methods

We established this systematic review according to the Preferred Reporting Items for Systematic Reviews and Meta-Analyses (PRISMA) guidelines. A protocol was developed and adopted by the medical affairs and ethics committee of our hospital.

### Search Strategy

Eligible studies were searched by two authors (J.L.G. and D.D.Z.) independently based on a previously designed search strategy. We searched the following electronic databases and websites for articles, study protocols and ongoing studies: Cochrane Central Register of Controlled Trials (CENTRAL), EMBASE, Medical Literature Analysis and Retrieval System Online (MEDLINE), Web of Science, Scopus and ClinicalTrials.gov. We also used Open Grey to search for grey literature.

The following search terms were used: [(Ovarian hyperstimulation syndrome) OR (OHSS)] AND Prevention AND ((albumin) OR (hydroxyethyl starch) OR (calcium) OR (glucocorticoid) OR (steroid) OR (metformin)]. Terms were adjusted in accordance with different database search rules. No publication date or language restrictions were set in this procedure.

### Study Selection

Two authors reviewed article titles and abstracts (J.L.G. and D.D.Z.) independently to select studies that met the inclusion criteria. Another author (Y.Z.) was consulted when there was any disagreement regarding study selection.

### Inclusion Eligibility

Studies that met the following criteria were included: (1) article type: RCTs reported by original articles or meeting papers; (2) patients: females undergoing COS in the process of assisted reproductive technology (ART); (3) interventions: pharmacologic therapies for the prevention of OHSS, including cabergoline, IV albumin, IV calcium, IV HES, aspirin, glucocorticoid, metformin or the combination of these therapies; intervention should be provided before the onset of clinical OHSS; (4) comparators: drugs as noted above, placebo or no intervention; and (5) outcome: OHSS incidence and post-intervention pregnancy rate.

### Exclusion Criteria

The following studies were excluded: (1) repeated reports; (2) reports without complete outcome data; (3) observational studies; (4) studies involving potential non-pharmacologic preventing methods, such as minimal stimulation protocols, coasting, targeted unifollicle induction, etc.; and (5) studies that initiated pharmacologic intervention after the onset of OHSS as a therapeutic method.

### Data Extraction and Quality Assessment

Study data were independently extracted by two authors (J.L.G. and Y.Z.) using a data collection form. Extracted data were verified by another author (X.M.Z.), and disagreements were resolved by an expert council.

Data extracted included study characteristics, primary outcome (OHSS incidence) and secondary outcome (clinical pregnancy rate). Original investigators were contacted by email when we had questions regarding their articles.

The Cochrane Risk of Bias assessment tool was applied independently by two authors (Y.S.Z. and Z.C.Q.) to assess the quality of the studies.

### Outcome Summary

The primary outcome, as noted above, was OHSS incidence, which means OHSS per randomized person. We preferred to extract the incidences of moderate to severe OHSS because clinicians paid more attention to these situations. When the moderate to severe OHSS incidence was unavailable, an overall rate that consisted of all levels of OHSS (mild, moderate, severe, critical) was adopted. Post-intervention clinical pregnancy was the secondary outcome. If a study article could not define the clinical pregnancy rate, we used the undefined pregnancy rate as an alternate. Study authors were contacted when there were unclear or undefined outcome data. If the author could not be reached, we adopted alternative outcome data, as described above.

Intension-to-treat (ITT) analysis was performed when studies contained participants who were lost to follow-up. We assumed that OHSS or clinical pregnancy did not occur to the lost participants. Additionally, if a study contained a high rate of follow-up loss, we tended to judge it as “High Risk” in the risk of bias assessment as missing unexplainable data.

### Statistical Analysis

NMA was performed when a closed evidence loop was available. A node-splitting analysis was adopted to test the consistency between direct evidence and indirect evidence for their agreement on a specific note (the split node)^11^. A Bayesian network meta-analysis based on Markov chain Monte Carlo methods using a consistency model was used to estimate the pooled RR values and the 95% credible intervals (CrIs) of the direct and indirect comparisons. This framework also allowed us to estimate the rank probability that each of the treatments was the best, the second best, etc. This entire procedure was performed using ADDIS software^12^ (v1.16, drugis.org, Groningen, The Netherlands).

Pair-wise direct head-to-head comparisons were performed to evaluate the pooled RR and 95% confidence interval (CI). Heterogeneity was estimated using the I^2^ statistic. I^2^ < 25% indicated a low risk of heterogeneity, and we then used a fixed-effects model to estimate the pooled outcome. Otherwise, when a high risk of heterogeneity was indicated, we used a random-effects model. This procedure was performed using Review Manager (RevMan v5.3, The Cochrane Collaboration, London, UK).

ITCs were performed when direct comparisons or closed evidence loops (which are essential to making MTCs) could not be achieved. We introduced a generalised linear regression model to estimate the ITC pooled RR and 95% CI. This procedure was performed using Stata (v12.0, StataCorp LP, Texas, USA).

Publication bias was assessed with a funnel plot and Begg’s rank correlation test^13^ using Stata (v12.0, StataCorp LP, Texas, USA).

## Results

### Study Selection

**Figure 1:**
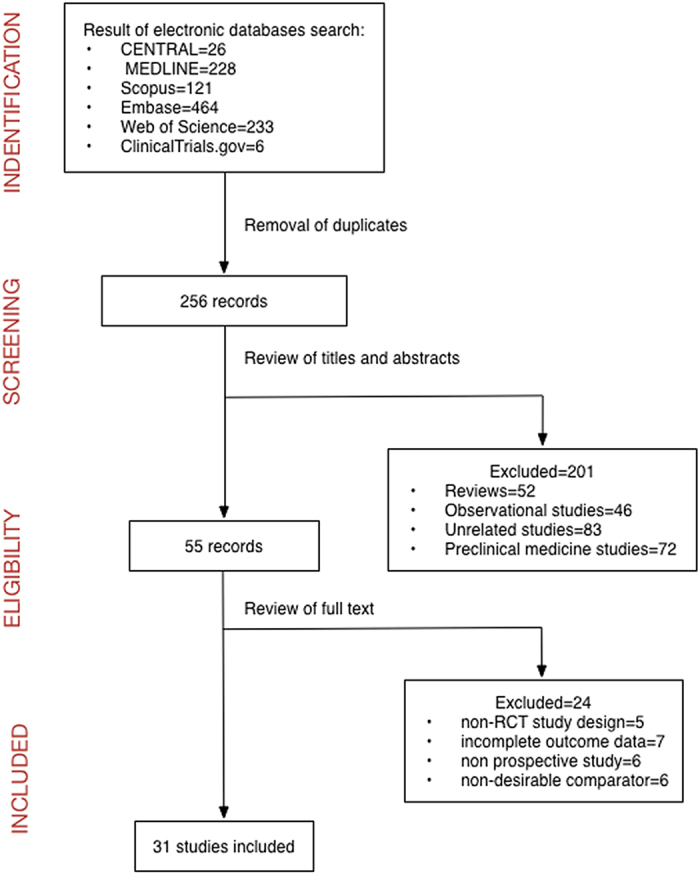
Flow diagram of the identification and selection of publications.

After the database search and the exclusion of duplicates, we enrolled 256 records for inspection. Two hundred one articles were excluded after the review of titles and abstracts. We excluded 52 reviews, 46 observational studies, 83 unrelated studies and 72 preclinical biomedical studies. Fifty-five records were left for further review of the full text. We continued to exclude 24 articles: 5 studies for non-RCT study design, 7 studies for incomplete primary outcome data, 6 studies because they were not prospective and another 6 studies for having a non-desirable comparator. Thirty-one studies^9^,^14^,^15^,^16^,^17^,^18^,^19^,^20^,^21^,^22^,^23^,^24^,^25^,^26^,^27^,^28^,^29^,^30^,^31^,^32^,^33^,^34^,^35^,^36^,^37^,^38^,^39^,^40^,^41^,^42^,^43^ were included for meta-analysis. A flowchart showing the process of study selection and inclusion is provided (Fig. 1).

### Study Baseline Characteristics and Quality Assessment

We summarised the main characteristics in Table 1.

Full-text articles could be accessed in 23 studies^9^,^16^,^17^,^18^,^21^,^22^,^24^,^26^,^27^,^28^,^29^,^30^,^31^,^32^,^34^,^36^,^37^,^38^,^39^,^40^,^41^,^42^,^43^, and other article types, including meeting papers^25^,^33^,^35^, abstracts^14^,^15^,^23^, letters^19^ and data from a previous meta-analysis^20^, were also included. We attempted to contact some of the authors for the full-text articles but received no responses.

All studies included were parallel-designed RCTs that originated from Europe, North America, Asia and the Middle East. Most of the studies were single centre except for 2 dual-centre and 1 multi-centre study. The dual-centre studies were from Saudi Arabia and Egypt^39^ and Italy and the USA^32^; the multi-centre study was from Norway, Denmark, Sweden and Finland^36^. Twenty-nine studies were 2 arm, and 2 studies were 3 arm^25^,^33^. The time of the clinical trial enrolment and article publications varied from 1992 to 2015. During this period of time, a total of 7181 participants were enrolled in the original studies and in this pooled analysis. The inclusion criterion of all included studies was participants at risk of developing OHSS. However, there was no precise definition of “risk of developing OHSS”. Therefore, investigators used the following rules to define the risk: (i) serum E_2_ level > 5505–14680 pmol/L (1500–4000 pg/L) on the day of hCG injection, (ii)>20 follicles detected by ultrasonography with a diameter>14 mm, and (iii)>20 oocytes retrieved. Nine studies enrolled patients with polycystic ovarian syndrome (PCOS) as high-risk candidates.

Classic long protocol and hCG triggers were adopted by all the included studies except for one article that used a short gonadotropin protocol and another six articles in which we could not obtain information concerning their COS protocol. The dose of hCG for triggering varied from 5000 to 10,000 IU. Interventions included 7 single-drug therapies (cabergoline, aspirin, IV albumin, IV HES, IV calcium, glucocorticoid, metformin) and 3 dual-drug therapies (cabergoline + IV HES, cabergoline+albumin). Comparators were placebo or no treatment (P/N) in 25 studies^9^,^14^,^15^,^16^,^17^,^18^,^19^,^20^,^21^,^22^,^23^,^24^,^25^,^26^,^27^,^28^,^29^,^30^,^32^,^33^,^34^,^35^,^36^,^37^,^38^ or one of the nine interventions in the remaining 6 studies^31^,^39^,^40^,^41^,^42^,^43^. The recipe for each medication is shown in Table 1.

All studies reported OHSS incidence as a primary outcome. The diagnosis and grading criteria of OHSS were not identical; 6 studies adopted Golan’s criteria^22^,^28^,^39^,^40^,^41^,^42^, 3 adopted Schenker’s^15^,^17^,^21^, 3 adopted Humaidan’s^9^,^37^,^38^, 2 adopted Navot’s^16^,^29^ and the remaining studies adopted self-defined criteria and WHO 1973 criteria. Twenty-three studies^9^,^15^,^16^,^17^,^19^,^20^,^21^,^22^,^23^,^24^,^26^,^27^,^29^,^31^,^32^,^34^,^36^,^37^,^38^,^39^,^40^,^41^,^43^ reported the pregnancy rate as one of the secondary outcomes. A definition of clinical pregnancy was provided in 9 articles^9^,^16^,^17^,^21^,^22^,^27^,^29^,^36^,^39^ as a positive β-hCG test 14 days after embryo transfer or the detection of foetal heart activity by ultrasonography, and the remaining studies did not provide their criteria. Study data extracted are shown in Supplementary Table A.

The risk of bias figure and the risk of bias summery figure are attached as Supplementary Figures A1-2. Overall, the quality assessment reviewed the included studies as having a low to moderate risk of bias.

### Network Meta-Analysis (NMA)

#### OHSS Incidence

**Figure 2:**
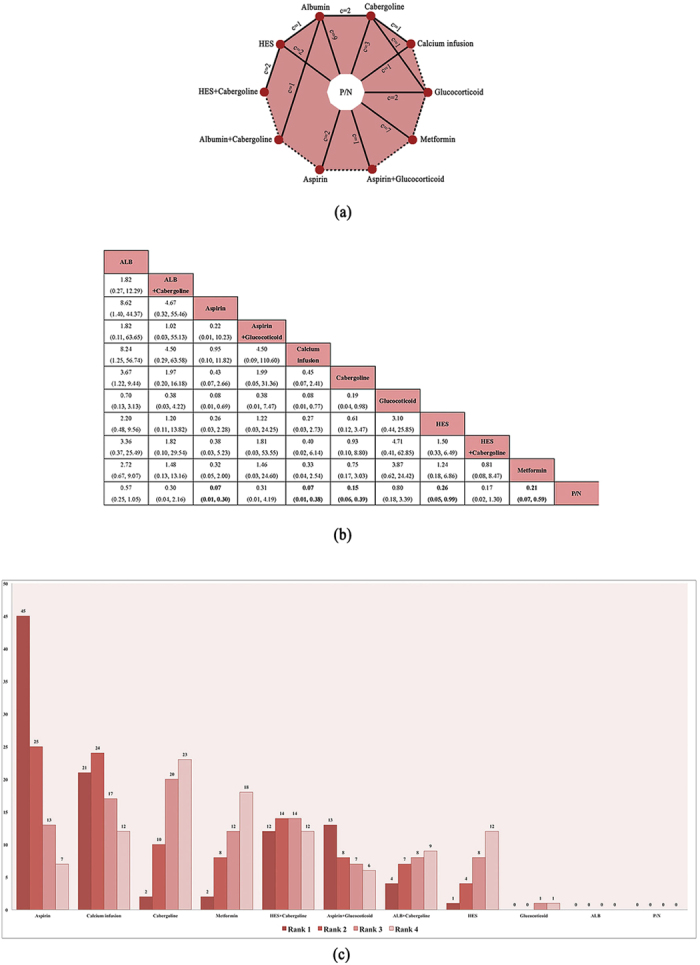
(**a**) Network figure of the evidence in the network meta-analysis for the primary outcome (OHSS incidence). Each red circle represents a pharmacological intervention. “c = n” represents the clinical trial number (including two 3-arm trials) between each comparator. Solid lines stand for the existence of direct evidence. Dotted lines represent no direct evidence. **(b)** Pooled relative risk and 95% credible interval (the numbers in parenthesis) of the primary outcome (OHSS incidence) based on the combination of direct and indirect evidence from the network meta-analysis. The longitudinal column interventions are compared with the transverse raw comparators. Bold numbers represents significant differences. **(c)** Rank probability figure for the interventions of network meta-analysis of the primary outcome (OHSS incidence) of ranks 1–5. A smaller rank number represents a better estimated effect. Each number (expressed in per cent) in this table demonstrates an estimated probability of an intervention to occupy a specific rank. Note. P/N = placebo or no treatment; HES = hydroxyethyl starch.

A closed-loop network was established and is presented in Fig. 2a. Based on this network, we performed NMA to combine the direct and indirect evidence for the pooled RR of OHSS incidence. The results are presented in Fig. 2b. Good coherence was confirmed from the combination of direct and indirect comparisons of the primary outcome, and the node-splitting test indicated no significant inconsistency (all p > 0.05). The test results are shown in Supplementary Table B.

Compared with P/N, 5 pharmacologic interventions were superior in decreasing OHSS incidence: aspirin (RR 0.07, 95% CrI 0.01–0.30, *p* < 0.05), IV calcium (RR 0.11, 95% CrI 0.02–0.54, *p* < 0.05), cabergoline (RR 0.17, 95% CrI 0.06–0.43, *p* < 0.05), metformin (RR 0.20, 95% CrI 0.07–0.59, *p* < 0.05) and IV HES (RR 0.26, 95% CrI 0.05–0.99, *p* < 0.05). Other interventions did not result in a significant difference compared with P/N. However, when compared with each other, aspirin, IV calcium, cabergoline and metformin did not show any difference in the significance level.

The rank gram (Fig. 2c) indicated the probability of being the best treatment, the second best, the third best, and so on among all the interventions for decreasing the OHSS incidence. Based on our network, the interventions having the probability of being the most effective were the following: aspirin (36%), IV calcium (35%), cabergoline (1%), metformin (1%) and HES (1%). Other interventions and their related rank probabilities were of less importance and were for reference only due to the result of the network meta-analysis. The cumulative rank probabilities are summarized in Supplementary Table C.

### Pair-Wise Direct Head-to-Head Comparisons

#### OHSS Incidence

**Figure 3:**
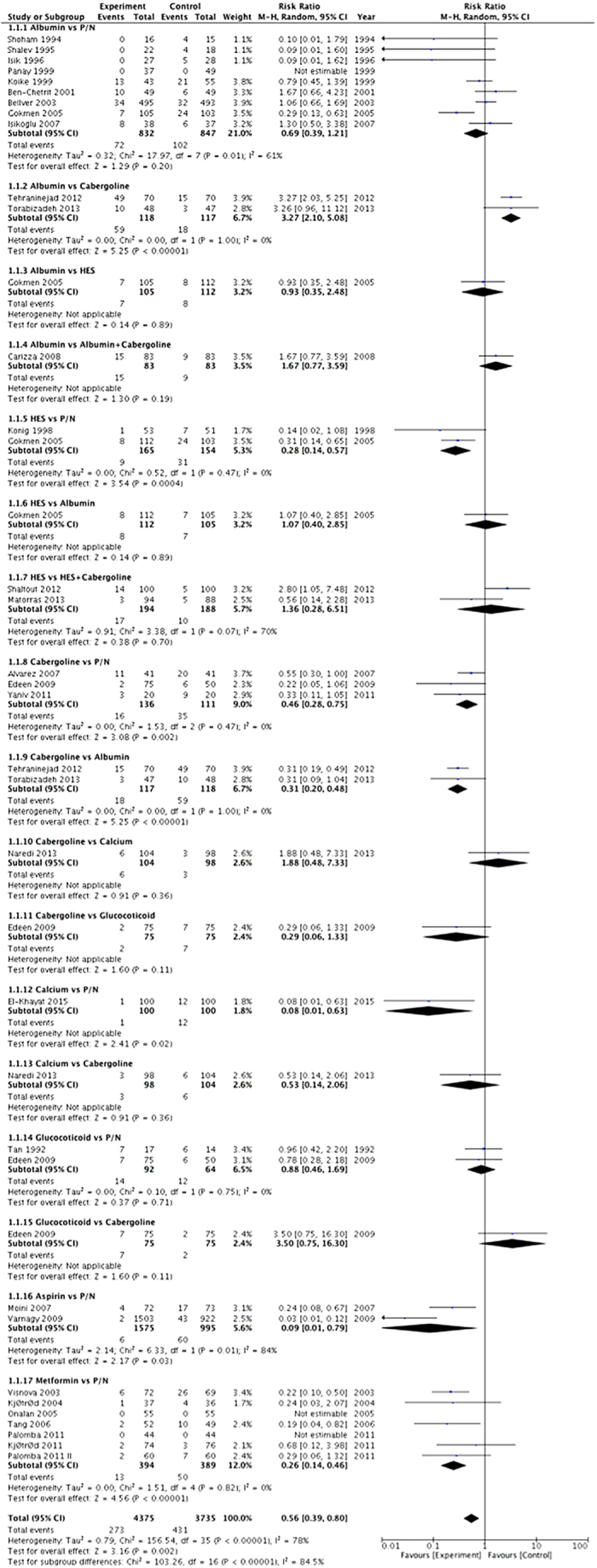
Forest plot of the direct pair-wise head-to-head comparisons between the interventions and their comparators for primary outcome (OHSS incidence). “Experiment” in the plot represents intervention, and “control” represents comparator.

Results from the direct comparison of the primary outcomes of the standard head-to-head meta-analysis are presented in Fig. 3.

Compared with P/N, 5 pharmacologic interventions were illustrated to be superior in preventing OHSS: aspirin (RR 0.09, 95% CI 0.01–0.79, *p* < 0.05), IV calcium (RR 0.08, 95% CI 0.01–0.63, *p* < 0.05), cabergoline (RR 0.46, 95% CI 0.28–0.75, *p* < 0.01), metformin (RR 0.26, 95% CI 0.14–0.46, *p* < 0.0001), and IV HES (RR 0.28, 95% CI 0.14–0.57, *p* < 0.001). Other direct comparisons did not show any significant difference except cabergoline vs. albumin (RR 0.31, 95% CI 0.20–0.48, *p* < 0.001).

#### Pregnancy Rate

Results from the direct comparisons of the secondary outcome of the standard head-to-head meta-analysis are presented in Supplementary Figure B.

With limited evidence, the direct comparison showed that albumin might decrease the pregnancy rate when compared with P/N (RR 0.85, 95% CI 0.74–0.97, *p* < 0.05). However, the results comparing albumin with comparators revealed no significant difference vs. cabergoline (RR 1.18, 95% CI 0.76–1.81, *p* = 0.47) and vs. albumin + cabergoline (RR 0.97, 95% CI 0.66–1.42, *p* = 0.87). The three other interventions demonstrated no difference compared with P/N: IV calcium (RR 0.91, 95% CI 0.73–1.13, *p* = 0.38), aspirin (RR 0.96, 95% CI 0.85–1.09, *p* = 0.54), and aspirin + glucocorticoid (RR 1.24, 95% CI 0.98–1.58, *p* = 0.07).

### Indirect Treatment Comparisons (ITCs)

#### Pregnancy Rate

Due to insufficient direct evidence, we performed ITCs to evaluate intra-intervention effectiveness. The results from the indirect comparisons of the secondary outcome by ITC are presented in Supplementary Table D. The ITC network is shown in Supplementary Figure C.

As the table illustrates, IV albumin was inferior to aspirin + glucocorticoid (RR 0.50, 95% CI 0.30–0.84, *p* < 0.05) and metformin (RR 0.56, 95% CI 0.38–0.83, *p* < 0.05) with respect to the pregnancy rate. However, other drug-drug ITCs did not show any significant differences.

### Quality of Evidence and Publication Bias

Heterogeneity was detected in several head-to-head meta-analyses (I^2^ > 50%). We adopted a random-effect model to minimise the influence of heterogeneity on our results.

The funnel plot and the Begg’s rank correlation test (Supplementary Figure D) detected no significant publication bias (Begg’s test, *p* = 0.285 > 0.05). However, the imbalance in the number of included studies for each intervention may contribute unreliability to the publication bias tests.

## Discussion

With the rapid development of assisted reproductive technology (ART) and the extensive use of controlled ovarian stimulation (COS), ovarian hyperstimulation status of variable severity occurs in nearly all women upon gonadotropin (Gn) administration and is characterised by the presence of ovarian enlargement due to multiple luteinized cysts within the ovaries and vascular hyperpermeability leading to ascites^44^. As a considerable health-threatening and costly iatrogenic complication of COS, the overall incidence of OHSS has exceeded 30%, and approximately 3–8% of cases are a critical and severe form due to hypovolaemia with oliguria, vast ascites, pleural effusion, and electrolyte imbalance. Thromboembolism complication and embolism are the most alarming complications of severe OHSS. Accordingly, we mainly focused on the moderate to severe OHSS incidence as a primary outcome in this systematic review. OHSS can be prevented to some extent at different stages. Primary preventive strategies, such as reducing the Gn dose and duration, applying a GnRH-antagonist protocol instead of the traditional luteal long protocol with pituitary down-regulation, and triggering ovulation using a GnRH-agonist rather than hCG, have been demonstrated to be valid^45^. Because of the specific restriction in certain circumstances and the dominant status for the GnRH-agonist long protocol in most parts of the world, the interventions described above cannot be applied across the board. Therefore, an adjuvant pharmacologic therapy is always considered to be a paramount complement for OHSS prevention. Different types of medicine are encountered in practice, but unfortunately, no standard recommendation for making clinical decisions currently exists. To the best of our knowledge, this is the first comprehensive network meta-analysis to compare the prophylactic effectiveness of OHSS among most of the popular published regimens and to estimate the influence of these treatments on the clinical pregnancy rate after ART treatment. Based on our results, as compared with placebo, aspirin, IV calcium, cabergoline, metformin and IV HES all work effectively in preventing OHSS without affecting the pregnancy rate, and these medicines are arranged in rank from the most effective to least effective. However, albumin is not effective in prevention and safe conception is not guaranteed, and thus this therapy should be avoided.

Vascular endothelial growth factor (VEGF) plays a leading role in increasing vascular permeability, which is the main pathophysiological process occurring in OHSS. Siess *et al.* and Chen *et al.* illustrated that this phenomenon is correlated with histamine, serotonin, platelet-derived growth factor, or lysophosphatidic acid (LPA) secreted by activated platelets^46^,^47^. Aspirin is a well-known nonsteroidal anti-inflammatory drug. Aspirin inhibits cyclooxygenase-1 (COX-1) in the platelet and results in an anti-platelet effect, which may disturb the pathological cascade driven by those cellular factors described above^34^. Earlier trials also provided evidence that low-dose aspirin treatment (100 mg) improved ovarian responsiveness, uterine and ovarian blood flow velocity, and implantation and pregnancy rates in patients undergoing *in vitro* fertilization^48^. According to our results, aspirin was the best prophylactic regimen for OHSS (RR 0.07, 95% CI 0.07–0.30, *p* < 0.05, Rank 1 36%, Rank 2 29%) and had no side effects with respect to the clinical pregnancy rate. Moreover, high-risk factors for embolism in OHSS patients include a non-physiologic high level of E2 itself during the COS and the hypovolaemia and condensation of blood cells. These risk factors would also benefit from the anti-thrombus activity of aspirin.

IV calcium is a relatively new method for preventing severe OHSS from occurring by inhibiting the renin-angiotensin system (RAS) and, consequently, reducing VEGF concentration. As far back as 1987, there was evidence that a significantly higher level of prerenin and renin were in the ovarian follicular fluid of Gn-stimulated ovaries compared with ovaries without Gn stimulation^49^. Other experiments showed that the difference in angiotensin II (Ang-II) levels between OHSS and non-OHSS ascites fluid was 100 times greater^50^. Ang-II could stimulate the release of VEGF locally, and fluid leakage induced by VEGF could promote RAS activity in turn^51^. With respect to the associated possible teratogenic effect, clinicians do not prescribe angiotensin converting enzyme inhibitors (ACEIs) and angiotensin receptor blockers (ARBs) to prevent OHSS^52^. Nevertheless, the study performed by Beierwaltes suggested that increased intracellular calcium might alter adenylyl cyclase and, thus, lead to a decreased level of cyclic adenosine monophosphate (cAMP), which means that calcium infusion was able to regulate the RAS in OHSS patients inversely^53^. Through the multiple treatment comparison among all RCTs included, IV calcium demonstrated superiority in the ranking list—Rank 1 (35%), Rank 2 (27%)—second only to aspirin. Considering its cost effectiveness and safety, we believe calcium is a relatively practical intervention in OHSS prevention.

Because VEGF is the most predominant mediator of the OHSS process, by targeting the VEGF receptor directly, it is possible to arrest this dangerous complication. Bates *et al.* demonstrated that the VEGF receptor 2 (VEGFR-2) should be blamed for the vascular hyperpermeability^54^,^55^. Theoretically, cabergoline, which is a dopamine agonist, can inhibit phosphorylation of the VEGFR-2 receptor and, therefore, reduce the vascular leakage into the third space and alleviate various presentations of OHSS after the COS cycle^56^. Based on the network meta-analysis, cabergoline significantly reduces the incidence of moderate to severe OHSS when compared with a placebo or blank control groups, and there is a 22% possibility that it is the most effective prophylactic drug among the five effective medical therapies described above. Notably, both cabergoline and calcium target the VEGF pathway, but the preventive mechanisms are slightly different: cabergoline antagonizes the VEGF-stimulating VEGFR-2, whereas calcium acts by reducing the VEGF level. Both treatments are acceptable in terms of effectiveness for OHSS prevention and safety for pregnancy; however, calcium is administered intravenously and cabergoline is more expensive.

Metformin belongs to the biguanide family and is an insulin-sensitising drug used to control hyperglycaemia in type 2 diabetes mellitus patients. In recent years, metformin has been recommended as the second-line treatment for oligo-ovulation or anovulation PCOS patients who are resistant to clomiphene citrate. In terms of ART treatment, metformin is prescribed in infertile PCOS patients or those whose glucose metabolism is impaired before or during COS, and it has been demonstrated to effectively reduce the number of nonperiovulatory follicles and E2 secretion by periovulatory follicles^37^. These effects lead to a decreased OHSS incidence in PCOS patients receiving COS, and all of the trials included here proved either neutral or positive with respect to the clinical pregnancy rate, which is in line with a recently published Cochrane review^8^. However, the following two facts are noteworthy. An RCT performed by Palomba *et al.* revealed that metformin worsened the ovarian response to Gn and decreased the MII oocyte number in females with reduced ovarian reserve^38^. Metformin administration is only accepted in PCOS or in some patients with abnormal glucometabolism and is currently not indicated for use in other infertile women at high risk of OHSS^57^. Because metformin is a category B drug and non-teratogenic, we suggest metformin can be a safe prevention strategy of OHSS for PCOS patients with normal or hyperovarian response undergoing assisted reproduction.

Because extravagant fluid leaks from the intravascular space and accumulates in the third compartment, the hallmark pathological change of OHSS is the massive blood volume depletion and haemoconcentration. Colloid infusion has been theorised to be an efficacious means of both symptomatic and aetiological treatment for this hypovolaemia and haemoconcentration condition, and the colloid molecules are thought to be able to deactivate the vasoactive mediators in OHSS^15^. Human albumin infusion is routinely used for the acute or chronic management of hypovolaemia and hypoproteinaemia and was first introduced as a preventive drug for severe OHSS in 1993^58^. Thereafter, the efficacy and safety of IV albumin for OHSS prevention have been constantly explored and questioned. Our literature search result revealed only one trial and meta-analysis that supported the positive effect of IV albumin in OHSS prevention [odds ratio (OR) 0.28, 95% CI (0.11–0.73)]^10^,^59^. In contrast, most of the original articles and meta-analyses oppose the prophylactic use of albumin due to dubious effectiveness and safety concerns^60^. Our network meta-analysis agreed with the results of most clinical trials. IV albumin, compared with placebo, has no significantly different effect in preventing OHSS (RR 0.57, 95% CI 0.25–1.05, *p* < 0.05), yet in terms of reducing the pregnancy rate, it is statistically significant(RR 0.85, 95% CI 0.74–0.97, *p* < 0.05). OHSS patients have a compromised vascular permeability; thus, the oncotic pressure generated by albumin may not be sufficiently sustained to prevent OHSS before the albumin itself leaks into the extravascular space^44^. A compromised pregnancy rate after IV albumin is explained by the albumin simultaneously binding other molecules operative in implantation as well as the vasoactive molecules in OHSS. Generally, the ordinary expression of, rather than eliminating, cytokines such as VEGF is essential in the conceiving process. Albumin is a blood-derived product and is therefore expensive and valuable in clinical practice. Unfortunately, albumin administration still possibly contributes to allergies and viral or prion infections of the recipients. These characteristics suggest that IV albumin should not be recommended for the routine prevention of OHSS. As an alternative, HES is much cheaper and is a non-biologically derived colloid fluid that is free from the risks noted above. HES works as a blood expander and prevents severe OHSS from occurring by increasing the oncotic pressure. Our results showed that HES was more effective than the placebo when compared with all prophylactic interventions included (RR 0.26, 95% CI 0.05–0.99, *p* < 0.05), which is consistent with a Cochrane review by Youssef *et al.* (OR 0.12; 95% CI 0.04–0.40)^60^. None of our calculations indicated that HES could hamper the pregnancy rate. From the perspective of cost-effectiveness and safety, HES is much more suitable as a pharmacologic strategy for OHSS prevention compared with albumin, but more evidence should be sought prior to the generalised routine use of this drug.

Other prophylactic agents, such as glucocorticoids (RR 0.80, 95% CI 0.18–3.39, *p* < 0.05) and combining therapies, such as albumin + cabergoline (RR 0.30, 95% CI 0.04–2.16, *p* < 0.05), HES + cabergoline (RR 0.17, 95% CI 0.02–1.30, *p* < 0.05) and aspirin + glucocorticoid (RR 0.31, 95% CI 0.01–4.19, *p* < 0.05), have not been demonstrated to be significantly from the network, but none of these treatments are harmful to the clinical pregnancy rate. Some results concerning combined therapies might not be strongly convincing because the number of subjects was not high in these trials. Further RCTs are expected to compare the effects of combined therapies, especially with drugs targeting different stages of the OHSS pathological process. The results of these RCTs may generate much more compelling results in the primary prevention of OHSS.

## Conclusions

Although no available pharmacologic intervention fully prevents the development of OHSS, adjuvant drug therapy can still be adopted to limit the incidence and improve the management of moderate to severe OHSS. According to our results, aspirin, IV calcium, cabergoline, IV HES and metformin have all been demonstrated to be efficient methods of preventing OHSS and have no effect on the pregnancy rate. Among the drugs listed above, aspirin and IV calcium are superior to the others in preventive effectiveness. Notably, metformin should serve as a prophylactic regimen mainly in PCOS patients without a poor ovarian reserve. However, IV albumin is neither effective in prevention nor does it guarantee safe conception, and this therapy should not be recommended. Our conclusion from this network meta-analysis may provide clinicians with an objective and comprehensive reference to guide their selection of pharmacologic intervention in OHSS prevention.

## Additional Information

**How to cite this article**: Guo, J.-L. *et al.* Pharmacologic Interventions in Preventing Ovarian Hyperstimulation Syndrome: A Systematic Review and Network Meta-Analysis. *Sci. Rep.* **6**, 19093; doi: 10.1038/srep19093 (2016).

## Supplementary Material

Supplementary Information

## Figures and Tables

**Table 1:** Characteristics of the included studies.

Study	Country of Origin; Time of Enrollment	Participants	COS Protocol	Intervention	Comparator
Tan 1992	The UK; NR	E2 > 10,000 pmol/mL and/or >20 follicles(>12 mm) on hCG day.	Long	Hydrocortisone 100 mg + Prednisolone 10 mg t.i.d.* 5 days + Prednisolone 10 mg b.i.d.* 3 days and 10 mg q.d.* 2 days^#^	No treatment
Shoham 1994	Israel; NR	E2 > 7,000 pmol/L and multiple follicular development.	NR	Albumin 50g i.v.^#^	Placebo^#^
Shalev 1995	Israel; NR	E2 > 9200 pmol/1 on the day of HCG, and >20 follicles >14 mm.	Long	Albumin 20gi.v.^#^	No treatment
Isik 1996	Turkey; July 1993–June 1994.	E2 > 11010 pmol/L on hCGday, combined with multifollicular response.	Long	Albumin 10gi.v.^#^	No treatment
Konig 1998	Germany; June 1996–Dec 1997.	E2 > 1500 pg/mL and/or >10 follicles onhCG day.	Long	6% HES solution 1000 mL i.v.^#^	Placebo^#^
Koike 1999	Japan; Jan 1996–Sept 1997.	≥20 oocytes retrieved.	NR	Albumin 37.5gi.v.q.d.* 3 days^#^	Placebo^#^
Panay 1999	NR; NR	E2 > 13000 pmol/L or 20 folicles.	NR	Albumin 20g i.v.^#^	No treatment
Ben-Chetrit 2001	Israel; April 1999–Dec 1999.	E2 > 10000 pmol/L or retrieval of >20 oocytes.	Long	Albumin 50g i.v.^#^	Placebo^#^
Visnova 2003	Czech; May 2000–Dec 2001.	PCOS patients.	Long	Metformin 500 mg b.i.d. from the first day of ovulation induction to OPU day.	No treatment
Bellver 2003	Spain; March 1999–Feb 2002.	Collection of >20 oocytes during oocyte retrieval.	Long	Albumin 40gi.v.^#^	No treatment
KjØtrØd 2004	Norway; Jan 2001 – June 2002	PCOS patients.	Long	Metformin 500 mg b.i.d. for at least 16 weeks ending on hCG day.	Placebo^*^
Gokmen 2005	Turkey; NR	E2 > 3000 pg/mL and/or >20 follicles(>14 mm) on hCG day.	NR	(i) 6% HES solution 50 mL^#^ (ii) 20% human albumin 50 mL^#^	Placebo^#^
Onalan 2005	Turkey; NR	PCOS patients.	Long	Metformin 850 mg b.i.d. or t.i.d. for 8 weeks before their first ICSI cycles, through the luteal phase, and until a positivepregnancy test.	Placebo*
Tang 2006	The UK; 2001–2004	PCOS patients.	Long	Metformin 850 mg b.i.d. from the start of the down-regulation process until the day of oocyte collection.	Placebo*
Alvarez 2007	Spain; April 2004–July 2006.	20–30 follicles larger than 12 mm and retrieval of more than 20 oocytes.	Long	Cabergoline 0.5 mgq.d.* 8 days^#^	Placebo^#^
Isikoglu 2007	Turkey; Jan 2003–Dec 2004.	E2 > 4,000 pg/mL or >20 follicles ≥ 14 mm onhCG day.	Long	Albumin 20gi.v.^#^	Placebo^#^
Moini 2007	Iran; April 2002–Jan 2004.	NR	Long	Aspirin 100 mg q.d.^#^ until menstruation or a negative pregnancy test	Placebo^#^
Carizza 2008	Brazil; Oct 2005–Sept 2007.	E2 > 4000 pg/mL.	Long	Cabergoline 0.5 mg q.d.* 21 days + Albumin 20g i.v.^#^	Albumin 20gi.v.^#^
Revelli 2008	Italy & the USA; Oct 2002–April 2006.	(i) IVF/ICSI, (ii) first treatment cycle, (iii) fresh ET, (iv) normal ovarian reserve.	Long	Aspirin 100 mg, from the 1st day of COS to the day of pregnancy test+ Predisolone5 mg b.i.d., from the 1st day of COS until the day before ET, 10 mg t.i.d., for 5 days starting from the day of ET; and 10 mg q.d.again, until the day of the pregnancy test.	No treatment
Varnagy 2009	Hungary; Jan 2000–Dec 2006.	All IVF patients	Long/GnRH-antagonist	Aspirin 100 mg q.d. started on the first day of the menstrual cycle when IVF was performed.	No treatment
Edeen 2009	United Arab Emirates; NR	PCOS patients	NR	Prednisolone 10 mg b.i.d.till the date of pregnancy test + Cabergoline0.5 mg q.d.* 2 days, repeated one week later.^†^	No treatment.
Yaniv 2011	Israel; NR	E2 > 4000 pg/ml on hCG day	NR	Cabergoline 0.5 mg q.d.* 8 days^#^	No treatment.
Palomba 2011	Italy; Jan 2009–Oct 2010.	(i) PCOS patients, (ii) age > 35 yr.	Short	Metformin 500 mg t.i.d., started1 month before COH, until a positive pregnancy test or menstrual bleeding occurred.	Placebo*
Palomba 2011 II	Italy; May 2009–Nov 2010.	(i) PCOS patients, (ii) previous cycle cancellation due to OHSS, (iii) age <= 35 yr.	Long	Metformin 500 mg t.i.d., started on the day of GnRH-agonist administration, until a positive pregnancy test or menstrual bleeding occurred.	Placebo*
KjØtrØd 2011	Norway, Denmark, Sweden, Finland; NR	PCOS patients.	Long	Metformin 500–2000 mg q.d. for the first 2 weeks of IVF/ICSI treatment.	Placebo*
Shaltout 2012	Saudi Arabia & Egypt; Jan 2007–Oct 2010.	E2 > 3500 pg/ml on hCG day, 20 follicles >12 mm, retrieval of more than 20 oocytes.	Long	Cabergoline 0.25 mg q.d.* 8 days^†^+ HES 500 ml i.v.^#^.	No treatment.
Tehraninejad 2012	Iran; June 2009–Dec 2010.	20–30 follicles >12 mm, and retrieval >20 oocytes.	Long	Cabergoline 0.5 mg q.d.* 7 days^#^	Albumin 20g i.v.^#^
Torabizadeh 2013	Iran; 2009.	>20 oocytes oocyte retrieved, ovary size >10 cm, E2 > 2500 pg/ml on hCG day.	Long	Cabergoline 0.5 mg q.d.* 8 days^†^	Albumin 20g i.v.^#^
Naredi 2013	India; Jan 2011–May 2015.	PCOS, >18 follicles >12 mm, history of OHSS.	Long	Cabergoline 0.5 mg q.d.* 8 days^†^	Calcium Gluconate1g i.v.* 3 days^#^
Matorras 2013	Spain; 2008–2009.	E2 > 3000, <5000 pg/mL and/or >20 follicles(>12 mm) on hCG day.	Long	6% HSE 500 mLi.v.^#^	Cabergoline 0.5 mg* 8 days^†^
El-Khayat 2015	Egypt; Oct 2011–Sept 2013.	Patients E2 on day of hCG > 2,500 pg/mLwith at least 20follicles >10 mm on the day of hCG administration.	Long	1 g Calcium gluconatei.v.* 3 days^#^	Placebo ^#^

Note. NR = not reported; i.v. = intravenous injection; q.d. = quaque die (once per day); b.i.d. = bis in die (twice per day); t.i.d. = ter in die (3 times per day); IVF = *in vitro* fertilization; ICSI = intra-cytoplasmic sperm injection; ET = embryo transfer; COS = controlled ovarian stimulation; OPU = ovum pick-up.

#Began on ovum pick-up (OPU) day. †Began on hCG day. *The same recipe as for the intervention.
