# Supplementary material for: Pharmacologic Interventions in Preventing Ovarian Hyperstimulation Syndrome: A Systematic Review and Network Meta-Analysis
Source: Sci Rep. 2016 Jan 11;6:19093. doi: 10.1038/srep19093 (PMC4707491; doi:10.1038/srep19093)
Supplement: Supplementary Information [file srep19093-s1.pdf]

Pharmacologic Interventions in Preventing Ovarian Hyperstimulation Syndrome: A Systematic Review and Network Meta-Analysis

Jun-Liang Guo[1,2,#], Duo-Duo Zhang[1,2,#], Yue Zhao[2,#], Dan Zhang[1], Xi-Meng Zhang[2], Can-Quan Zhou[1,*], Shu-Zhong Yao[1,*]

1 Department of Obstetrics & Gynaecology, The First Affiliated Hospital of Sun Yat-sen University, Guangzhou City, 510080, P.R. China

2 Zhong-shan School of Medicine, Sun Yat-sen University, Guangzhou City, 510082, P.R. China

* Corresponding Author A: Shu-Zhong Yao, M.D., Chief Professor of the Department of Obstetrics & Gynaecology, The First Affiliated Hospital of Sun Yat-sen University, Guangzhou City, 510080. yszsysu@163.com. Corresponding Author B: Can-Quan Zhou, M.D., Chief Professor of the Centre of Reproductive Medicine, The First Affiliated Hospital of Sun Yat-sen University, Guangzhou City, 510080; zhoucanquan@hotmail.com.

# These authors contributed equally to this work.

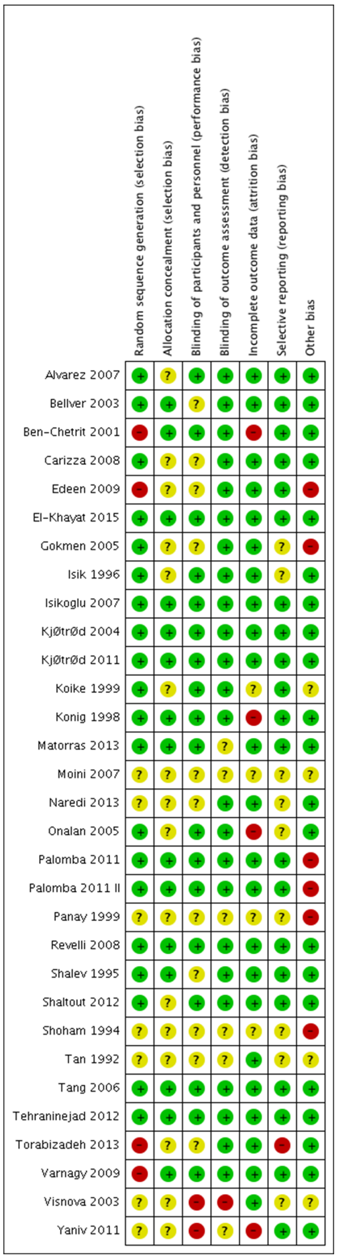

1

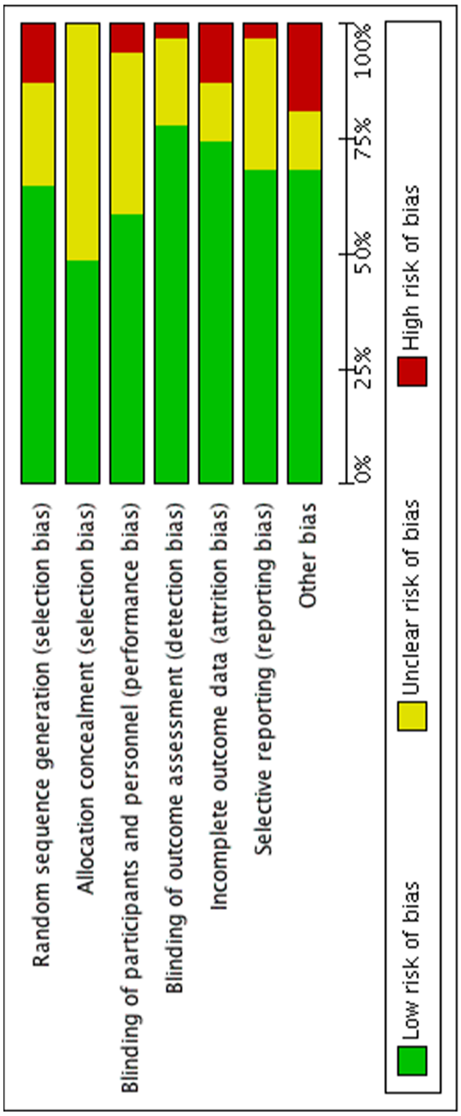

2

Supplementary Figure A.

1. Risk of bias summary figure of included studies. 2. Risk of bias figure of included studies.

Study or Subgroup	Experiment Events	Experiment Total	Control Events	Control Total	Weight	Risk Ratio M-H, Random, 95% CI	Year
**2.2.1 Albumin vs P/N**							
Shoham 1994	4	16	2	15	0.4%	1.88 [0.40, 8.78]	1994
Shalev 1995	6	22	2	18	0.5%	2.45 [0.56, 10.72]	1995
Isik 1996	5	27	5	28	0.8%	1.04 [0.34, 3.18]	1996
Koike 1999	14	43	24	55	3.1%	0.75 [0.44, 1.26]	1999
Panay 1999	11	31	28	45	3.1%	0.57 [0.34, 0.97]	1999
Ben-Chetrit 2001	14	49	16	49	2.5%	0.88 [0.48, 1.59]	2001
Bellver 2003	138	298	165	307	12.9%	0.86 [0.73, 1.01]	2003
Isikoglu 2007	21	38	23	37	5.1%	0.89 [0.61, 1.30]	2007
**Subtotal (95% CI)**		**524**		**554**	**28.4%**	**0.85 [0.74, 0.97]**	
Total events	213		265				
Heterogeneity: Tau^2 = 0.00; Chi^2 = 5.66, df = 7 (P = 0.58); I^2 = 0%							
Test for overall effect: Z = 2.42 (P = 0.02)							
**2.2.2 Calcium vs P/N**							
El-Khayat 2015	59	100	65	100	10.1%	0.91 [0.73, 1.13]	2015
**Subtotal (95% CI)**		**100**		**100**	**10.1%**	**0.91 [0.73, 1.13]**	
Total events	59		65				
Heterogeneity: Not applicable							
Test for overall effect: Z = 0.87 (P = 0.38)							
**2.2.3 Aspirin vs P/N**							
Varnagy 2009	463	1503	295	922	15.0%	0.96 [0.85, 1.09]	2009
**Subtotal (95% CI)**		**1503**		**922**	**15.0%**	**0.96 [0.85, 1.09]**	
Total events	463		295				
Heterogeneity: Not applicable							
Test for overall effect: Z = 0.62 (P = 0.54)							
**2.2.4 Aspirin+Glucocorticoid vs P/N**							
Revelli 2008	49	97	121	298	9.2%	1.24 [0.98, 1.58]	2008
**Subtotal (95% CI)**		**97**		**298**	**9.2%**	**1.24 [0.98, 1.58]**	
Total events	49		121				
Heterogeneity: Not applicable							
Test for overall effect: Z = 1.78 (P = 0.07)							
**2.2.5 HES vs HES + Cabergoline**							
Shaltout 2012	41	100	42	100	6.3%	0.98 [0.70, 1.36]	2012
Matorras 2013	48	94	43	0		Not estimable	2013
**Subtotal (95% CI)**		**194**		**100**	**6.3%**	**0.98 [0.70, 1.36]**	
Total events	89		85				
Heterogeneity: Not applicable							
Test for overall effect: Z = 0.14 (P = 0.89)							
**2.2.6 Albumin vs Cabergline**							
Tehraninejad 2012	26	70	20	70	3.6%	1.30 [0.80, 2.10]	2012
Torabizadeh 2013	5	10	2	3	0.9%	0.75 [0.27, 2.06]	2013
**Subtotal (95% CI)**		**80**		**73**	**4.6%**	**1.18 [0.76, 1.81]**	
Total events	31		22				
Heterogeneity: Tau^2 = 0.00; Chi^2 = 0.96, df = 1 (P = 0.33); I^2 = 0%							
Test for overall effect: Z = 0.73 (P = 0.47)							
**2.2.7 Albumin vs Albumin+Cabergoline**							
Carizza 2008	32	83	33	83	5.2%	0.97 [0.66, 1.42]	2008
**Subtotal (95% CI)**		**83**		**83**	**5.2%**	**0.97 [0.66, 1.42]**	
Total events	32		33				
Heterogeneity: Not applicable							
Test for overall effect: Z = 0.16 (P = 0.87)							
**2.2.8 Metformin vs P/N**							
Visnova 2003	17	72	10	69	1.8%	1.63 [0.80, 3.31]	2003
KjØtrØd 2004	15	37	14	36	2.7%	1.04 [0.59, 1.84]	2004
Onalan 2005	16	55	22	55	3.1%	0.73 [0.43, 1.23]	2005
Tang 2006	20	52	8	49	1.8%	2.36 [1.15, 4.85]	2006
Palomba 2011 II	13	44	16	44	2.5%	0.81 [0.45, 1.48]	2011
Palomba 2011	37	74	25	76	4.9%	1.52 [1.03, 2.25]	2011
KjØtrØd 2011	26	60	24	60	4.4%	1.08 [0.71, 1.66]	2011
**Subtotal (95% CI)**		**394**		**389**	**21.2%**	**1.18 [0.89, 1.56]**	
Total events	144		119				
Heterogeneity: Tau^2 = 0.06; Chi^2 = 11.05, df = 6 (P = 0.09); I^2 = 46%							
Test for overall effect: Z = 1.17 (P = 0.24)							
**Total (95% CI)**		**2975**		**2519**	**100.0%**	**0.99 [0.90, 1.10]**	
Total events	1080		1005				
Heterogeneity: Tau^2 = 0.01; Chi^2 = 30.48, df = 21 (P = 0.08); I^2 = 31%							
Test for overall effect: Z = 0.10 (P = 0.92)							
Test for subgroup differences: Chi^2 = 10.99, df = 7 (P = 0.14), I^2 = 36.3%							

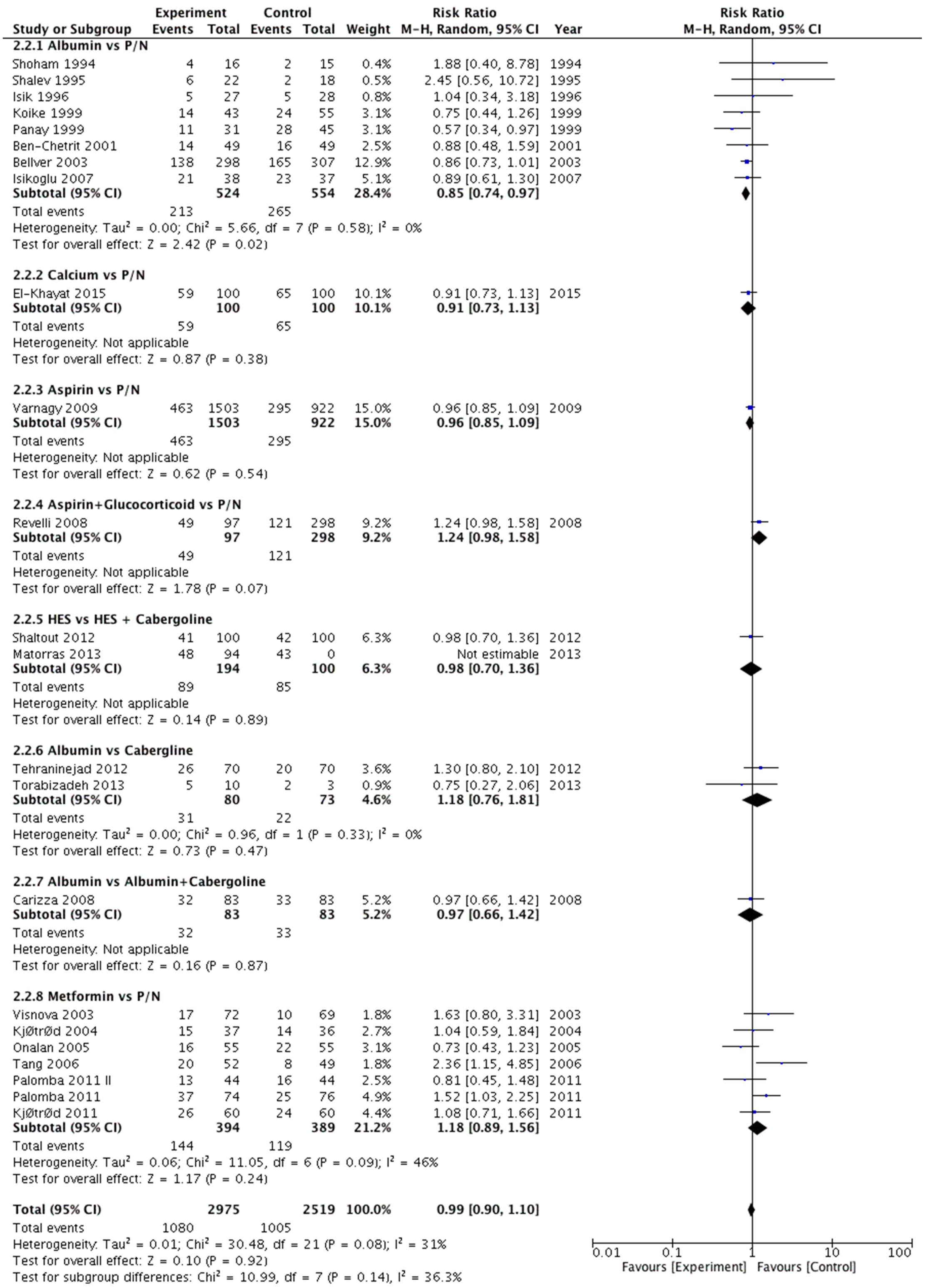

Supplementary Figure B.

Forest plot of the direct pair-wise head-to-head comparisons between interventions and their comparators for the secondary outcome (pregnancy rate). “Experiment” in the plot represents the intervention, and “control” represents the comparator.

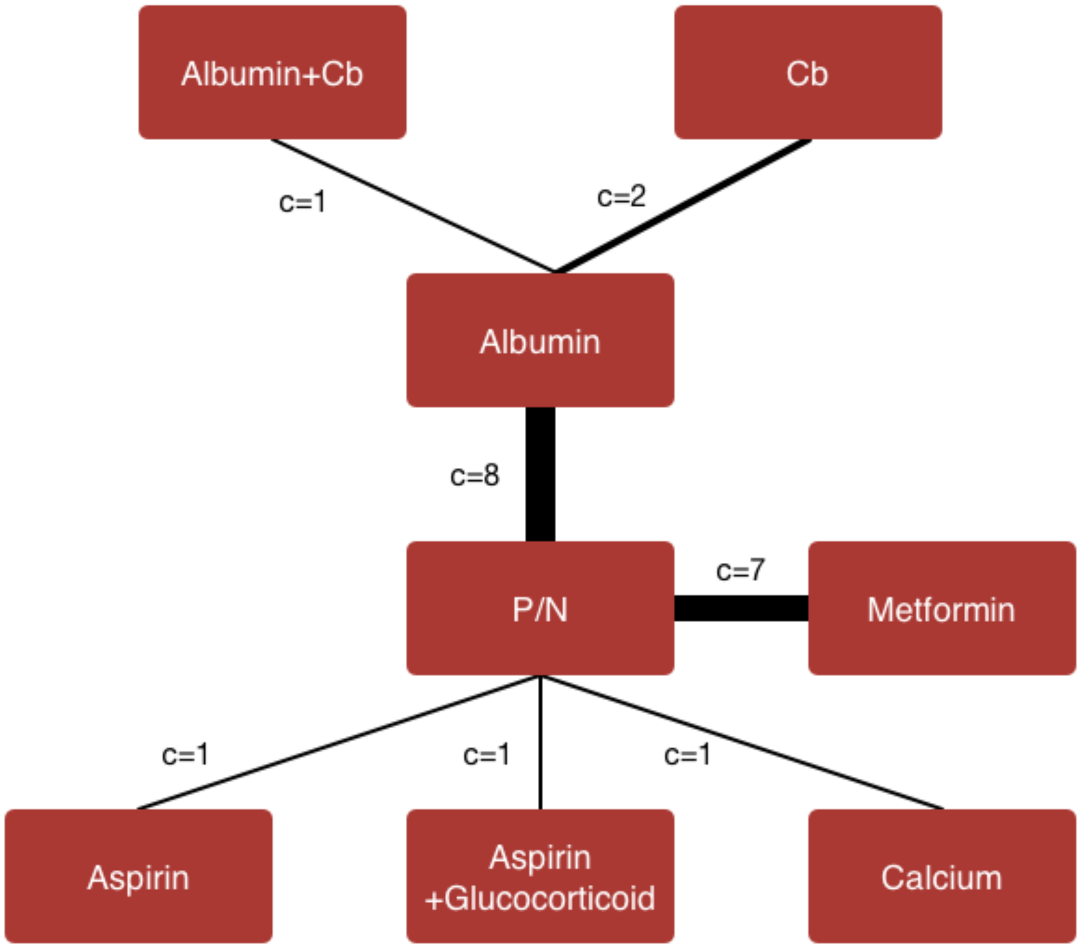

Supplementary Figure C. Network figure of evidence in the indirect treatment comparison for the secondary outcome (pregnancy rate). Each red rectangle represents a pharmacological intervention. "c=n" and the thickness of black columns represent the clinical trial number between each comparator.

Note: Cb=cabergoline; P/N=placebo or no treatment.

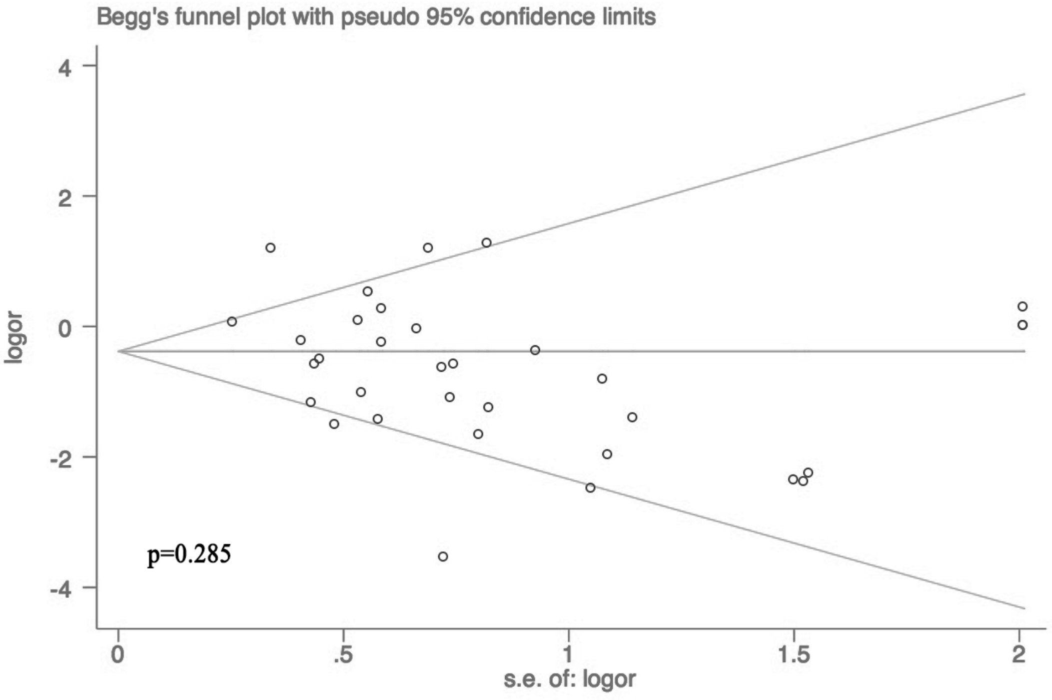

Supplementary Figure D.

Begg’s funnel plot and Begg’s regression test *p* value of included studies.

Study	Intervention<br>Comparator	OHSS incidence					Pregnancy rate		
		Mild OHSS	Moderate-Severe OHSS	Total OHSS	ITT Participant	Actual Participant	Pregnancy	ITT Participant	Actual Participant
Tan 1992	Glucocorticoid	NR	NR	7	17	17	NR	NR	NR
	P/N	NR	NR	6	14	14	NR	NR	NR
Shoham 1994 *	ALB	0	0	0	NR	16	4	NR	16
	P/N	0	4	4	NR	15	2	NR	15
Shalev 1995 *	ALB	0	0	0	22	22	6	22	19
	P/N	0	4	4	18	18	2	18	17
Isik 1996 *	ALB	0	0	0	27	27	5	27	27
	P/N	0	5	5	28	28	5	28	28
Konig 1998	HES	NR	1	1	53	51	NR	NR	NR
	P/N	NR	7	7	51	50	NR	NR	NR
Koike 1999 *	ALB	NR	13	13	43	43	14	43	43
	P/N	NR	21	21	55	55	24	55	55
Panay 1999 *	ALB	NR	NR	0	NR	37	11	NR	31
	P/N	NR	NR	0	NR	49	28	NR	45
Ben-Chetrit 2001 *	ALB	36	10	46	49	46	14	49	46
	P/N	35	6	41	49	41	16	49	41
Visnova 2003 *	Metformin	NR	NR	6	72	72	17	72	72
	P/N	NR	NR	26	69	69	10	69	69
Bellver 2003 *	ALB	NR	34	34	495	488	138	298	NR
	P/N	NR	32	32	493	488	165	307	NR
KjØtrØd	Metformin	NR	NR	1	37	31	15	37	31

2004 *	P/N	NR	NR	4	36	32	14	36	32
Gokmen 2005 *	HES	NR	8	8	112	112	NR	NR	NR
	ALB	NR	7	7	105	105	NR	NR	NR
	P/N	NR	24	24	103	103	NR	NR	NR
Onalan 2005 *	Metformin	3	0	3	55	53	16	55	53
	P/N	4	0	4	55	55	22	55	55
Tang 2006 *	Metformin	NR	2	2	52	47	20	52	47
	P/N	NR	10	10	49	47	8	49	47
Alvarez 2007	Cabergoline	NR	11	11	41	35	NR	NR	NR
	P/N	NR	20	20	41	32	NR	NR	NR
Isikoglu 2007 *	ALB	NR	8	8	38	38	21	38	38
	P/N	NR	6	6	37	37	23	37	37
Moini 2007	Aspirin	NR	NR	4	72	72	NR	NR	NR
	P/N	NR	NR	17	73	73	NR	NR	NR
Carizza 2008 *	Cabergoline +ALB	NR	NR	9	83	83	33	83	83
	ALB	NR	NR	15	83	80	32	83	80
Revelli 2008 *	Aspirin+ Glucocorticoid	NR	1	1	97	97	49	97	97
	P/N	NR	7	7	298	298	121	298	298
Varnagy 2009 *	Aspirin	NR	2	2	1503	1503	463	1503	1503
	P/N	NR	43	43	922	922	295	922	922
Edeen 2009	Glucocorticoid	NR	NR	7	75	75	NR	NR	NR
	Cabergoline	NR	NR	2	75	75	NR	NR	NR

	P/N	NR	NR	6	50	50	NR	NR	NR
Yaniv 2011	Cabergoline	NR	3	3	20	18	NR	NR	NR
	P/N	NR	9	9	20	19	NR	NR	NR
Palomba 2011 *	Metformin	0	0	0	44	44	13	44	44
	P/N	2	0	2	44	44	16	44	44
Palomba 2011 II *	Metformin	3	2	5	60	60	26	60	60
	P/N	11	7	18	60	60	24	60	60
KjØtrØd 2011 *	Metformin	NR	2	5	74	74	37	74	74
	P/N	NR	3	5	76	75	25	76	75
Shaltout 2012 *	Cabergoline +HES	5	5	10	100	100	42	100	100
	HES	7	14	21	100	100	41	100	100
Tehraninejad 2012 *	ALB	NR	49	49	70	69	26	70	69
	Cabergoline	NR	15	15	70	69	20	70	69
Torabizadeh 2013 *	ALB	NR	10	10	48	48	5	10	10
	Cabergoline	NR	3	3	47	47	2	3	3
Naredi 2013	Calcium	6	3	9	98	98	NR	NR	NR
	Cabergoline	10	6	16	104	104	NR	NR	NR
Matorras 2013 *	HES		NR	3	94	94	48	94	94
	Cabergoline +HES		NR	5	88	88	43	88	88
	P/N	NR	1	1	33	33	19	33	33
El-Khayat	Calcium	6	1	7	100	100	59	100	100

2015 *	P/N	11	12	23	100	100	65	100	100

Supplementary Table A. Summary of the extracted data of two outcomes from 31 studies.
Note: NR=not reported; P/N=placebo or no treatment.
* Pregnancy rate (secondary outcome) available.

Intervention	Comparator	Direct Effect		Indirect Effect		*p* value
		LRR	95%CI	LRR	95%CI	
Albumin	Cabergoline	-1.90	(-3.30, -0.47)	-0.69	(-2.01, 0.78)	0.19
Albumin	HES infusion	-0.17	(-2.09, 1.95)	-1.94	(-5.69, 0.81)	0.28
Albumin	P/N	0.71	(-0.02, 1.69)	0.20	(-1.44, 1.98)	0.54
Calcium infusion	Cabergoline	0.72	(-1.50, 3.09)	1.07	(-1.70, 4.71)	0.84
Calcium infusion	P/N	2.99	(0.40, 6.62)	2.59	(0.08, 5.19)	0.82
Cabergoline	Glucocorticoid	1.49	(-0.82, 3.88)	1.78	(-0.68, 4.41)	0.86
Cabergoline	P/N	1.41	(0.22, 2.71)	2.33	1.06, 3.76)	0.27

Supplementary Table B. Results of the node-splitting analysis for the assessment of inconsistencies. The analysis compares the direct effect with the indirect effect between interventions to estimate inconsistencies in the network analysis. *P* value>0.05 represents no significant difference between the direct and indirect effect.

Note: LRR=log relative risk; CI=confidence interval; P/N=placebo or no treatment.

Drug	Rank 1	Rank 2	Rank 3	Rank 4	Rank 5	Rank 6	Rank 7	Rank 8	Rank 9	Rank 10
ALB	0	0	0	0	1	5	13	29	36	14
ALB+Cabergoline	4	6	7	9	10	12	15	15	9	6
Aspirin	36	29	15	8	5	3	2	1	0	0
Aspirin+Glucocoticoid	12	8	7	6	8	8	9	10	9	8
Calcium infusion	35	27	15	9	5	4	2	1	1	0
Cabergoline	1	8	22	25	20	13	7	3	0	0
Glucocoticoid	0	0	1	1	2	4	8	13	17	23
HES	1	3	7	12	16	20	20	13	6	2
HES+Cabergoline	10	13	14	13	13	12	9	6	4	3
Metformin	1	5	11	17	19	19	15	8	3	1
P/N	0	0	0	0	0	0	0	3	13	42

Supplementary Table C. Rank probability result for interventions of the network meta-analysis of the primary outcome (OHSS incidence). The smaller rank number represents the better estimated effect. Each number (expressed in per cent) in this table demonstrates an estimated probability of an intervention to occupy a specific rank.

Note: P/N=placebo or no treatment.

Intervention	Comparator	Relative Risk	95% CI	*p* value
Albumin+Cb	P/N	0.78	[0.40,1.53]	0.473
Cb	P/N	0.54	[0.26,1.12]	0.099
Albumin	Aspirin	0.79	[0.58,1.07]	0.121
Albumin	Aspirin +Glucocorticoid	0.50	[0.30,0.84]	0.009
Albumin	Metformin	0.56	[0.38,0.83]	0.004
Albumin	Calcium infusion	0.96	[0.51,1.79]	0.897
Aspirin	Aspirin +Glucocorticoid	0.63	[0.39,1.04]	0.07
Aspirin	Calcium infusion	1.22	[0.67,2.22]	0.513
Aspirin+ Glucocorticoid	Calcium infusion	1.93	[0.92,4.02]	0.08
Metformin	Aspirin	1.40	[0.99,1.99]	0.057
Metformin	Aspirin +Glucocorticoid	0.89	[0.51,1.54]	0.678
Metformin	Calcium infusion	1.72	[0.90,3.28]	0.102

Supplementary Table D. Results of indirect treatment comparisons (ITCs) based on a generalised linear regression model for the secondary outcome (pregnancy rate).

Note: Cb=cabergoline; CI=confidence interval; P/N=placebo or no treatment.
